# Ionic Conductive Polymers for Electrochemical Devices

**DOI:** 10.3390/polym14020246

**Published:** 2022-01-07

**Authors:** Riccardo Narducci

**Affiliations:** 1 Department of Industrial Engineering, University of Rome “Tor Vergata”, 00133 Rome, Italy; riccardo.narducci@uniroma2.it; 2 International Laboratory Ionomer Materials for Energy (LIME), 00133 Rome, Italy

Increasing levels of pollution (especially in large cities), the rising cost of oil, and climate change are pushing the scientific community towards more sustainable solutions for the conversion and storage of energy. Devices such as fuel cells (FCs), redox flow batteries (RFBs), and electrolyzers can help to significantly decrease the amount of greenhouse gases emitted. Ionic conductive polymers are fundamental components of these devices (protonic, anionic, and amphoteric), generally requiring great chemical and mechanical stability; good performance and durability; low permeability to reagents; and excellent characteristics of weight, volume, and current density for several applications from mobile to automotive and co-generation systems. Unfortunately, the high cost of perfluorinated ionomers and the low stability of anionic polymers in alkaline environment, among other things, still limit their use. This Special Issue of *Polymers* is dedicated to this exciting research field, with some excursions in related fields, focusing on commercial polymers such as Nafion, a benchmark for proton conducting membranes, acid doped polybenzimidazole (PBI), or blended membranes containing hyperbranched PAES/Linear PPO as anion exchange membranes (AEMs). Promising and low-cost sulfonated aromatic polymers (SAP), such as sulfonated poly(ether ether ketone) (SPEEK) and sulfonated poly(phenyl sulfone) (SPPSU) ionomers in phosphate buffer solution for enzymatic fuel cell, or crosslinked sulfonated polyphenylsulfone-vinylon (CSPPSU-vinylon) are presented. The ecofriendly poly(vinyl alcohol) (PVA) used to modify the catalyst layer, or the use of novel ionic liquid-incorporated Zn-ion conducting polymer electrolyte membranes, or solid polymer blend electrolytes (SPBEs) based on natural chitosan (CS) and methylcellulose (MC) are proposed. In this book, we also report some strategies to enhance the mechanical stability, such as cross linking (XL), or several techniques, including classical casting methods or electrospinning (ES), used to obtain ionomer nanofibers with different morphologies depending on relative humidity (RH); all properties were studied with classical investigations techniques, such as impedance, dynamic mechanical analysis (DMA), FC tests, or the new INCA method. To reflect the broad scope of this topic, contribution from leading scientist across the world, whose research addresses ionomeric membranes and catalysts from different perspectives, sharing a common vision of pollution reduction and the search for sustainable energy sources, have been gathered. In this manner, Lufrano et al. [1] investigated how Nafion 1100 membrane preparation procedures affect both the morphology of the polymeric film and the proton transport properties of the electrolyte. A comparison between commercial membranes such as Nafion 117 and Nafion 212 and Nafion membranes prepared by three different procedures, Nafion-recast, Nafion uncrystallized, and Nafion 117-oriented, was conducted. The conductivity measurements show that the anisotropy increased from ~20% in the commercial membrane up to 106% in the pressed membrane (Nafion 117-oriented) where the ionic clusters were averagely oriented parallel to the surface, leading to a strong directionality in proton transport. The solution cast Nafion uncrystallized membrane showed the lowest water diffusion coefficients, conductivities, and Young’s modulus, highlighting the correlation between low crystallinity and a more branched and tortuous structure of hydrophilic channels. The authors suggested to avoid the use of both materials, oriented and uncrystallized, in high-temperature fuel cells. Pasquini et al. [2] studied the hydrolytic stability, conductivity, and mechanical behavior of SPEEK and SPPSU ionomers in phosphate buffer solution for enzymatic fuel cell. The results showed that the membrane stability can be adapted by changing the casting solvent (water, ethanol or DMSO) and procedures. A shorter casting time resulted in stiffer membranes but had no effect on the mass uptake (MU) and conductivity. The crosslinking stabilized the membranes that showed a better hydrolytic stability, even if with a little decrease of conductivity. The addition of SPPSU to SPEEK membranes improved the ionic conductivity. Kim et al. [3] prepared a series of novel AEMs with hyperbranched brominated poly(arylene ether sulfone) (Br-HB-PAES) and linear chloromethylated poly(phenylene oxide) (CM-PPO) with different weight ratios followed by quaternization with triethylamine, which promoted the ion channel formation. In particular, the Q-PAES/PPO-55 membrane showed a very high hydroxide ion conductivity (0.91 × 10^−1^ S cm^−1^) around three times higher than the pristine Q-HB-PAES membrane. In addition, the rigid hyperbranched structure showed an enhancement of the swelling ratio and demonstrated an alkaline stability under 2M KOH conditions over 1000h at 50 °C. Jienkulsawad et al. [4] employed, in the fabrication of membrane electrode assemblies (MEAs), a PVA to humidify the membrane in proton-exchange membrane fuel cells (PEMFCs) operated under low-humidity conditions. The 0.03 wt% PVA in the anode catalyst layer (CL) and 0.1 wt% PVA on the gas diffusion layer (GDL) improve the current density by approximately 30% at the operating voltage of 0.6 V and non-humidified anode and cathode humidifier temperature of 25 °C. Liu et al. [5] prepared a novel membrane containing a polymer matrix poly(vinylidene fluoride-hexafluoropropylene) (PVdF-HFP) and 1-ethyl-3-methylimidazolium trifluoromethanesulfonate (EMITf), along with zinc trifluoromethanesulfonate Zn(Tf)_2_. The best amorphous and nanopored membrane, ILPE-Zn-4, with a mass ratio of 0.4:0.4:1 (EMITf:Zn(Tf)_2_:PVDF-HF), showed at room-temperature (RT) ionic conductivity of ~1.44 × 10^−4^ S cm^−1^ with a wide electrochemical stability window (~4.14 V) and thermal decomposition temperature ~305 °C with good tensile strength ~5.7 MPa. This polymer electrolyte could be a promising candidate for energy storage applications. Aziz et al. [6] studied and synthesized, with a solution cast technique, SPBEs materials based on CS and MC incorporated with different concentrations of ammonium fluoride (NH_4_F) salt. The electrochemical stability of the electrolyte sample was found to be up to 2.3 V via the linear sweep voltammetry (LSV) study. The value of specific capacitance was determined to be around 58.3 F g^−1^. The synthesized electrical double-layer capacitor (EDLC) cell was found to exhibit high efficiency (90%). In the first cycle, the values of internal resistance, energy density, and power density of the EDLC cell were determined to be 65 Ω, 9.3 Wh kg^−1^, and 1282 W kg^−1^, respectively. Escorihuela et al. [7] presented a systematic study of the physicochemical properties and proton conductivity of PBI membranes doped with common phosphoric acid at different concentrations, 0.1, 1, and 14M, and with other alternative acids such as natural phytic acid (0.075M) and phosphotungstic acid (HPW, 0.1M). The cross-section SEM images show the formation of channels in the polymeric network thanks to the use of these acids, also keeping their mechanical properties and thermal stability. Under low acid doping (0.1M), membranes doped with phytic acid displayed a superior conducting behavior (2.6 × 10^−4^ S cm^−1^), compared to doping with phosphoric acid (5.8 × 10^−6^ S cm^−1^), proved to be a sustainable alternative. Kim et al. [8] synthesized a thermally crosslinked sulfonated polyphenylsulfone (CSPPSU) polymer and different wt% of PVA (5, 10, and 20); then, a CSPPSU-vinylon membrane was synthesized using a formalization reaction. The conductivity of the CSPPSU-10vinylon membrane reached 0.66 × 10^−1^ S cm^−1^ at 120 °C under 90% RH and was higher than CSPPSU membrane. The fuel cell results showed higher current densities than those of Nafion 212 and CSPPSU membranes, obtained under high- and low-humidification conditions. These results are due to the excellent water retention even under low humidification conditions of vinylon membranes and can be proposed as an alternative to fluoropolymer electrolytes, especially for thin membranes. Halabi et al. (Dekel’s group) [9] prepared, by ES technique, anion-conducting ionomer-based nanofibers with different morphologies depending on the RH during the process (RH_ES_). The formation of branched thin fibers was observed in mats prepared at RH_ES_ 20% and 30%. This affects the water uptake (WU) and conductivity, which are higher for fibers formed at low humidity, attributable to their larger diameter. The understanding of these parameters is important for the future design of these devices with tailored properties. Raja Rafidah et al. [10] discussed the recent progress of aromatic-based membranes that represent some of the best alternatives in proton exchange membranes (PEMs) due to their electrochemical, mechanical, and thermal strengths. Membranes based on these polymers, such as poly(aryl ether ketones) (PAEKs) and polyimides (PIs), however, lack a sufficient level of proton conductivity and durability. Various strategies are proposed to improve these characteristics: crosslinking, multiblock copolymerization, the introduction of inorganic/organic fillers/nanofillers, etc. Although they have disadvantages that limit their use in fuel cells, aromatic-based polymers still hold great potential as effective and low-cost alternatives to perfluorinated ones.

I am confident that the articles contained in this Special Issue will serve to further stimulate advances in this research area, in both the sectors of membranes and catalysts; the first is essential for the long-term functioning of the system, and the second for a drastic reduction in costs, especially in fuel cells. I thank all my friends and colleagues who contributed papers to this Special Issue.
